# Supplementary material for: Evaluation of Clinical Results regarding Peristomal Skin Health Associated with the Adjustment and Formulation of the New Moderma Flex One-Piece Ostomy Devices
Source: J Pers Med. 2023 Jan 26;13(2):219. doi: 10.3390/jpm13020219 (PMC9962063; doi:10.3390/jpm13020219)
Supplement: Supplementary file 1 [file jpm-13-00219-s001.zip › jpm-2003447-supplementary.pdf]

ANEX A

DATA COLLECTION NOTEBOOK

Information of the health professional (at the end of the questionnaire the Data Protection Notice is attached)

Name surname:
Hospital: Telephone:
E-mail:

Instructions: To fill in using survey monkey. The agreement of the health professional to fill out this product evaluation will be collected through legal text and "click" according to the legal parameters used in these cases.

FIRST CALL

Patient's clinical situation

1. **¿Has the patient been recently operated or a patient with an existing stoma?**

   Reciently operated ☐

   Existing stoma ☐

   In case of being a patient with an existing ostomy, indicate the time since the intervention::

   <3 months ☐ 3-6 months ☐ 6-12 months ☐

2. **Age of the patient:** ____________ years old

3. **Sex:**

   Male ☐ Female ☐

4. **Type of ostomy practiced::**

   Colostomy ☐ Permanent ☐ Terminal ☐

   Ileostomy ☐ Temporary ☐ Loop ☐

   Urostomy ☐

5. **Morphology of the stoma::**

   Round ☐ Oval ☐ Irregular shape ☐

   Protruded ☐ Flat ☐ Invaginated ☐

6. **Abdomen type:**

   Smooth ☐ Folds ☐ Scars ☐

   Globular ☐ Flaccid ☐

7. **Skin Type:**

   Dry ☐ Oily ☐ Mixed ☐

   Intact/healtht ☐ Irritated ☐ Damaged ☐

8. **Does the diagnosed patient have any peristomal skin complication?**

Irritative dermatitis ☐ Maceration ☐ Fungus ☐

Folicullitis ☐ Traumatic dermatitis ☐ Pressure ulcer ☐

Mucocutaneous separation ☐ Pioderma gangrenosum ☐ Others ☐

Observations on skin condition and complications::

____

9. **Does your patient have any condition that could put the health of the peristomal skin at risk?:**

Observations:____

(Answer only in the case of a patient with an existing ostomy)

10. **Device you are currently using:**

1 piece ☐ 2 pieces ☐

Closed ☐ Drainable ☐ Urostomy ☐

Flat barrier ☐ Convex barrier ☐

Brand/Reference: ____

11. **Accessories used in conjunction with the device:**

None ☐ Paste ☐ Rings ☐ Belt ☐ Powder ☐

Adhesive remover spray/wipes ☐ Derma protective wipes ☐

Others (specify): ____

12. **How often does the patient change their usual device? (Select only one):**

More than once a day ☐ Every day ☐ Every 2 days ☐

Every 3 days ☐ Every 4 days ☐ Every 5 days ☐

Every 6 days ☐ Every 7 days ☐ + 7 days ☐

13. **Reason for device change:**

Routine ☐ Leakage ☐ Irritation ☐

Itching ☐ Barrier peel off ☐ Barrier erosion ☐

Others (specify): ____

After use of Moderma Flex one-piece ostomy appliancess

Second call

1. **Moderna Flex device evaluated:**

   Closed ☐ Drainable ☐ Urostomy ☐

   Flat barrier ☐ Convex barrier ☐ Soft convexity ☐

   Adhesive border ☐ Display window ☐

   Reference: ____

2. **Accessories used in conjunction with the evaluated Moderma Flex device:**

   None ☐ Paste ☐ Rings ☐ Belt ☐ Powder ☐

   Adhesive remover spray/wipes ☐ Derma protective wipes ☐

   Others (specify): ____

3. **Frequency of change of the evaluated device (Select only one):**

   More than once a day ☐ Every day ☐ Every 2 days ☐

   Every 3 days ☐ Every 4 days ☐ Every 5 days ☐

   Every 6 days ☐ Every 7 days ☐ + 7 days ☐

4. **Reason for changing the evaluated device:**

   Routine ☐ Leakage ☐ Irritation ☐

   Itching ☐ Barrier peel off ☐ Barrier erosion ☐

   Others (specify): ____

5. **Rate the condition of the skin after using the tested device:**

   Much worse ☐ Worsen ☐ Same ☐

   Improved ☐ Very improved ☐

   Describe the condition of the skin after barrier removal:

   ____

6. **Only in the case of having evaluated a device with an adhesive border, report if the adhesive border has produced:**

   Allergy ☐ Irritation ☐ Itching ☐ None ☐

7. **Only in relation to the skin layer, rate the functioning and performance of the evaluated device (1 strongly disagree – 5 strongly agree):**

	1	2	3	4	5
Skin barrier flexibility	☐	☐	☐	☐	☐
Peristomal área adaptability	☐	☐	☐	☐	☐
Adaptability to abdomen	☐	☐	☐	☐	☐
Barrier peristomal fit	☐	☐	☐	☐	☐
Barriere adherence (barriers maintains adhered)	☐	☐	☐	☐	☐
Resistance to output erosion	☐	☐	☐	☐	☐
Ease of barrier application	☐	☐	☐	☐	☐
Smoothness in the removal of the barrier	☐	☐	☐	☐	☐
Barrier comfort	☐	☐	☐	☐	☐
Barrier safety	☐	☐	☐	☐	☐
Overall barrier performance	☐	☐	☐	☐	☐

8. **Only if you have evaluated the urostomy device, rate the degree to which the device works (1 strongly disagree – 5 strongly agree):**

	1	2	3	4	5
Comfortable to use	☐	☐	☐	☐	☐
Safe	☐	☐	☐	☐	☐
Discreet (multichamber mechanism)	☐	☐	☐	☐	☐
Easy to use (drain valve)	☐	☐	☐	☐	☐
Easy to open/close (drain valve)	☐	☐	☐	☐	☐
Easy to empty (drain valve)	☐	☐	☐	☐	☐

9. **Only if you have evaluated a device with a viewing window, please indicate the degree to which you have found it useful in terms of (1 not at all useful – 5 very useful):**

	1	2	3	4	5
Facilitate barrier placement	☐	☐	☐	☐	☐
Inspect the stoma condition	☐	☐	☐	☐	☐
Inspect the efluent contained in the pouch	☐	☐	☐	☐	☐
Provide greater discretion	☐	☐	☐	☐	☐

10. Final remarks regarding the performance of the evaluated device:
